# Scaling up the evaluation of psychotherapy: evaluating motivational interviewing fidelity via statistical text classification

**DOI:** 10.1186/1748-5908-9-49

**Published:** 2014-04-24

**Authors:** David C Atkins, Mark Steyvers, Zac E Imel, Padhraic Smyth

**Affiliations:** 1 Department of Psychiatry and Behavioral Sciences, University of Washington, 1100 NE 45th St., Ste. 300, Seattle, WA USA; 2 Department of Cognitive Sciences, University of California, Irvine, Irvine, CA USA; 3 Counseling Psychology Program, Department of Educational Psychology, University of Utah, Salt Lake City, UT USA; 4 Department of Computer Science, University of California, Irvine, Irvine, CA USA

**Keywords:** Motivational interviewing, Provider fidelity, Statistical text classification

## Abstract

**Background:**

Behavioral interventions such as psychotherapy are leading, evidence-based practices for a variety of problems (*e.g.*, substance abuse), but the evaluation of provider fidelity to behavioral interventions is limited by the need for human judgment. The current study evaluated the accuracy of statistical text classification in replicating human-based judgments of provider fidelity in one specific psychotherapy*—*motivational interviewing (MI).

**Method:**

Participants (n = 148) came from five previously conducted randomized trials and were either primary care patients at a safety-net hospital or university students. To be eligible for the original studies, participants met criteria for either problematic drug or alcohol use. All participants received a type of brief motivational interview, an evidence-based intervention for alcohol and substance use disorders. The Motivational Interviewing Skills Code is a standard measure of MI provider fidelity based on human ratings that was used to evaluate all therapy sessions. A text classification approach called a labeled topic model was used to learn associations between human-based fidelity ratings and MI session transcripts. It was then used to generate codes for new sessions. The primary comparison was the accuracy of model-based codes with human-based codes.

**Results:**

Receiver operating characteristic (ROC) analyses of model-based codes showed reasonably strong sensitivity and specificity with those from human raters (range of area under ROC curve (AUC) scores: 0.62 – 0.81; average AUC: 0.72). Agreement with human raters was evaluated based on talk turns as well as code tallies for an entire session. Generated codes had higher reliability with human codes for session tallies and also varied strongly by individual code.

**Conclusion:**

To scale up the evaluation of behavioral interventions, technological solutions will be required. The current study demonstrated preliminary, encouraging findings regarding the utility of statistical text classification in bridging this methodological gap.

## Background

Various forms of psychotherapy are among the most common and effective therapies for drug and alcohol problems [1], and hence, hundreds of thousands to millions of Americans are receiving some form of psychotherapy for alcohol and drug problems each year. For example, in 2010 the Substance Abuse and Mental Health Services Administration (SAMHSA) documented over 1.8 million treatment episodes for drug and alcohol problems [2], and separately, the Veterans Administration estimated that over 460,000 veterans were receiving care related to a substance use disorder [3]. What is the quality of these interventions? How do we evaluate them?

With pharmacotherapy, the medication quality is governed by the Food and Drug Administration and the therapeutic dosage and administration is specified by the drug manufacturer and can be tracked via electronic medical records. With psychotherapy, the quality of the intervention occurs at the time of the intervention and is fundamentally a part of the linguistic exchange between the patient and provider. For example, research on motivational interviewing (MI), an evidence-based psychotherapy for drug and alcohol problems, suggests that specific types of linguistic exchanges (*e.g.*, reflections, open questions) are related to positive patient outcomes [4]. Such findings have informed standards for proficient delivery of MI and have influenced national dissemination efforts [5].

Generally, provider fidelity has been defined as *‘*…the degree to which an intervention was implemented as it was prescribed in the original protocol or as it was intended by the program developers*’* (p. 69) [6]. The implementation field has noted a primary challenge associated with assessing provider fidelity to behavioral interventions—the requirement of direct human observation and judgment [6]. The reliance on human judgments for fidelity ratings leads to a fundamental gap between methods of assessing provider fidelity and the volume of care being delivered. As a result, it is impossible to evaluate provider fidelity in disseminated treatments in any ongoing way. The current study examines a novel methodology for automating the evaluation of provider fidelity in MI.

For decades, the research gold standard for evaluating provider fidelity has been observational coding—applying a theory-driven coding system to identify relevant behaviors and language in therapists. Behavioral coding requires training a team of human raters, establishing inter-rater reliability among the raters, and then performing the time-consuming task of coding. Although software can facilitate the coding process [7], human coding does not *‘*scale up*’* for dissemination in any meaningful sense. The time to code 100 hours of therapy is roughly ten times the amount for 10 hours of therapy, and as noted above, the actual number of alcohol and drug abuse sessions in the U.S. healthcare system run into the hundreds of thousands, if not millions, per year. In addition to clinical dissemination, research that has focused on studying treatment mechanisms (*e.g.*, identifying active ingredients of behavioral interventions) has struggled with similar methodological limitations. The typical size of psychotherapy mechanism studies is small due to behavioral coding demands, which contributes to incredible heterogeneity across studies examining the association of therapist behaviors with patient outcomes [8].

The current interdisciplinary research described in this paper is pursuing a technological solution for scaling up the evaluation of provider fidelity in MI, as well as other linguistically based coding systems. Our research draws on advances in statistical text analysis, specifically topic models [9-12], which were developed in computer science and have only recently been applied to psychotherapy data [13]. The present analyses used a recent extension of these approaches called the labeled topic model [14] that is well suited for psychotherapy transcripts with behavioral coding data. In particular, the model can predict codes at the level of talk turns and overall sessions, which map on to therapy mechanism research and provider fidelity ratings, respectively. The present research represents a preliminary, proof of concept study, focused on the goal of computer-based coding of MI intervention transcripts (*i.e.*, generate observational codes of psychotherapy without humans).

## Method

### Addiction corpus

The present research used 148 sessions from five MI studies [15-19], representing a random sample of the total number (n = 899 sessions) available. Table 1 summarizes the intervention studies and shows the number of sessions, talk turns, and overall word count across the intervention studies. Although all five studies included MI in one or more treatment arms, they are relatively heterogeneous in other characteristics. Three of the studies were treatment development focused (ESP21, ESPSB, iCHAMP), whereas HMCBI was an effectiveness study, and ARC was an efficacy trial. The university-based studies predominantly used graduate or undergraduate students as providers, who received training and weekly supervision, whereas HMCBI relied primarily on clinic-based social workers to deliver the MI, with monthly group supervision.

**Table 1:** Description of the addiction corpus comprised of five motivational interviewing studies

**Study**	**Description**	**Sessions**	**Talk turns**	**Words**^f^
ARC^a^	Brief alcohol intervention for first year college students with some indication of drinking related problems.	10	1,768	72,712
ESPSB^b^	Brief alcohol intervention for students intending to drink during their upcoming spring break trip.	20	5,266	182,738
ESP21^c^	Brief alcohol intervention for students turning twenty-one years old.	41	9,967	391,164
HMCBI^d^	Brief drug intervention for adults presenting at primary care clinics who indicate drug use.	70	11,039	284,097
iCHAMP^e^	Brief marijuana intervention for college students with some indication of marijuana-related problems	7	1,950	74,213
Total		148	29,990	1,004,924

^a^Alcohol Research Collaborative: Peer Programs [19].

^b^Event Specific Prevention: Spring Break [17].

^c^Event Specific Prevention: Twenty First Birthday [18].

^d^Brief Intervention for Problem Drug Use and Abuse in Primary Care [15].

^e^Indicated Marijuana Prevention for Frequently Using College Students [16].

^f^Excludes punctuation.

Each of the sessions was transcribed and coded using the Motivational Interviewing Skills Code (MISC) [20]. The current methods use text as their basic input and hence require transcription; we discuss the issue of transcription as a potential barrier to these methods in the Discussion. Details on the statistical models are below, but the basic linguistic representation in our analysis focuses on the set of words in each talk turn often referred to as *‘*n-grams*’* in the statistical text analysis literature, including individual words (unigrams) as well as combinations of words involving two or three words (bigrams and trigrams).

### MISC coding

A modified version of the MISC 2.1 [20] was used to code each transcript. The MISC is a coding system for provider and patient utterances that identifies MI consistent (*e.g.*, complex reflections, empathy) and inconsistent (*e.g.*, closed questions, confrontation) provider behaviors, and patient language related to changing or maintaining their drug or alcohol use. Each human coder segmented talk turns into utterances (*i.e.*, complete thoughts) and assigned one code per utterance for all utterances in a session. The majority of sessions were coded once by one of three coders (79%; n = 117). To assess inter-rater reliability, 21% (n = 31) of sessions were coded by all three coders. Reliability of human coders is reported below along with reliability of statistical text classification methods. The present analyses focused on the 12 MISC codes that were present in 2% or more of talk turns. Note that one modification of the current MISC coding was that human raters indicated talk turns that were characteristic of empathy and MI spirit. Traditionally, these are considered global codes and rated once for an entire session. Present analyses used these talk-turn codes for empathy and MI spirit, which allowed a single labeled topic model to be fit to all codes.

### Topic models and prediction tasks

A topic model is a machine-learning model for text [10]. Given a set of documents, or other text such as transcripts, a topic model will estimate underlying dimensions of the linguistic content, called topics. A topic is represented as a distribution over words, and documents are represented as distributions over topics. Thus, an individual session is modeled as a mixture of topics, where each topic represents a cluster of words. The current research used a variant of the topic model that incorporates coded data, or more generally, types of meta-data that are outside the texts themselves [14]. Meta-data is a general term in machine learning that refers to data that provides additional information or descriptors of another dataset. With session transcripts, meta-data is simply any non-transcript data and could be coding data, as in the present application, or self-report data (*e.g.*, severity of drug use) or demographic information (*e.g.*, gender or socioeconomic status). Machine learning also broadly divides models into supervised models, in which a model learns associations from inputs (*i.e.*, predictors) to an outcome (*e.g.*, logistic regression is considered a supervised method), and unsupervised models, in which a model is discovering unknown groups in the data (*e.g.*, cluster analysis is an unsupervised learning method). The labeled topic model used in the current analyses is semi-supervised in that the model directly learns which text is associated with which codes, in addition to a number of ‘background’ topics that are not associated with any codes and account for linguistic variance unassociated with specific MISC codes.

In evaluating the prediction accuracy of topic models to generate MISC codes, the current research used a 10-fold cross-validation procedure in which the 148 sessions were randomly divided into 10 equal partitions. The accuracy of the model is then established by training the labeled topic model on 90% of sessions and testing the accuracy on the remaining 10% of sessions that were held out of training. This procedure then iterates 10 times and the model predictions on the test sessions are combined across partitions for an overall accuracy estimate on all 148 sessions. Importantly, the model accuracy is always based on testing predictions on new sessions that the model did not have access to during training. For the training sessions that were coded by three coders, the model used the union of codes across raters as *‘*truth.*’* Therefore, if the three raters applied codes A + B, B + C, and D, respectively to a particular talk turn (indicating great uncertainty about its content), the model assumed that the talk turn was labeled with codes A, B, C, and D. (Further methodological details on how the analyses were conducted and evaluated are contained in the Additional file 1: Supplemental Appendix).

## Results

### Inferred topics

Prior to assessing prediction accuracy of model-based MISC codes, we descriptively examined the topics generated by the model (Table 2). For each topic, the top 20 terms with highest probability are shown. Three types of topics were specified in the model, corresponding to individual MISC codes, study (*i.e.*, a topic for each unique intervention study), and a set of background, or general, topics. The inferred topics associated with MISC codes and intervention studies are generally interpretable, which is a typical feature and strength of topic models. For example, questions (QU) are associated with word combinations one expects of questions (what do you, do you think). Reflections (RE) are associated with fragments that are common to many reflections (sounds like).

**Table 2:** Most likely word and word phrases for topics associated with MISC codes study type and background

**Label type**	**Label**	**High probability words and word combinations**
MISC Codes	QUC (Question Closed; T)	have you, questions, would you, are you, risk, drink, you think, okay so, do you have, heard, interested, drinking, drinks, do you think, sound, you have, use, is that, study, does that, sounds, questions about, you ever, called, have you ever, you say, would you say, any questions about, any questions
	QUO (Question Open; T)	what do you, what do, group, do you think, you think, expected, how do you, bar, students, study, how do, what are, tell me, groups, how does, thoughts, looked, you know about, get alcohol, so what, how does that, drinking, questions do you, you make of, expect, what are your, do you know, drink, do you make
	RES (Reflection Simple; T)	sounds, it sounds like, mentioned, okay so, sounds like, it sounds, like you, you said, you were, sounds like you, drinking, drink, you mentioned, okay so you, you were saying, noticed, alcohol, you said you, so it sounds, were saying, earlier, said you, what you, talked, strategies, drinks, you said that, you mentioned that, limit, experience
	REC (Reflection Complex; T)	sounds, it sounds like, sounds like, it sounds, sounds like you, like you, so it sounds, important, okay so, for you, you have, okay so you, drink, drinking, with your, alcohol, you were, experience, like you have, you’re really, drinks, you really, it seems, school, kind of, seems like, important to you, responsible, you do, responsibilities
	AF (Affirm; T)	great, appreciate, thank, thanks, thank you, that’s good, that’s great, excellent, for coming in, alcohol, thank you for, drink, for coming, coming in, you for, appreciate you, drinking, glad, drinks, surveys, you have, i appreciate, questions, thanks for, nice, study, sounds, we really appreciate, taking the time, great so
	GI (Giving Information; T)	alcohol, blood, drinks, hours, b a c, students, a c, chart, based, weight, looks, level, content, typical, blood alcohol, risk, asked, occasion, it looks like, a point, blood alcohol content, this is, number, drinking, drink, peak, card, point oh, stomach, look at
	ST (Structure; T)	survey, feedback, information, section, drink, alcohol, page, we’ll, alright, with you, packet, drinking, talked, we can, so we, move, talk about, little bit about, some of, we’re gonna, conversation, based, bit about, the next, give you, start, surveys, personalized, what you, check
	SU (Support; T)	alcohol, drink, drinking, sounds, drinks, difficult, hope, tough, it sounds like, i hope, kind of, a lot, need, less, a lot of, friends, it sounds, use, for you, spring, i know, i think, you’ve been, you do, sounds like, effects, hours, you know
	E (High Empathy; T)	kind of, for you, sounds, hand, it seems like, drinking, alcohol, what you, you do, seems like, drink, like you, okay so, it seems, friends, drinks, mentioned, where you, fit, your friends, helps, fun, social, it sounds like, sounds like, with your, something that you, it sounds, you have, hearing
	SP (High Spirit; T)	questions, interested, drinking, information, things that you, alcohol, asked, drink, for you, what you, things that, some of, thoughts, use, goals, looking, about your, tell me, what are, kind of, you like, plans, the things, students, interested in, more about, of the things, are some of, okay so, things you
	+ (Change Talk; C)	when i, drink, quit, drinking, like i, money, don’t wanna, can’t, i would, alcohol, don’t like, stop, it i, need, i can’t, don’t want, i can, where i, know i, i ca, high, i know, i have, i need to, mean i, i know i, i mean i, stupid, weed, sick
	- (Sustain Talk; C)	smoke, when i, weed, i’m not, drink, i mean, smoking, i have, like i, i would, i feel, i feel like, it’s not, feel like, drinking, mean i, alcohol, i can, i like, it i, sit, drunk, i smoke, fun, high, buy, relax, don’t have, for me, i mean i
Study Type	ARC	alcohol, drinking, drink, calories, positive, negative, drinks, level, a lot of, you’re drinking, effects, point oh, percent, excellent, sounds, blackout, beer, common, all right, dependence, beers, relaxed, hour, instances, tend, that point, man, typical, effect, average
	ESPSB	break, spring, drinks, spring break, sex, standard, drink, student, average, number, drinking, u w, students, over spring break, typical, on spring break, plans, what you, alcohol, trip, your spring break, u w student, for spring break, mixed, over spring, actual, ounces, island, numbers, vegas
	ESP21	birthday, drinks, wine, drink, standard, beer, alcohol, your birthday, twenty first birthday, bar, drinking, mixed, glass, shots, twenty first, bars, dinner, for your birthday, ounces, twenty one, first birthday, celebration, researchers, on your birthday, how many, dancing, iced, standard drinks, mixed drinks, groups
	HMCBI	drug, you know, years, pain, using, drugs, you know what, know what i, marijuana, you know i, i’m saying, doctor, i’ve been, call, treatment, quit, what i, crack, i can’t, i’m not, old, phone, cocaine, use, i got, drug use, i said, ai, clean, depression
	iCHAMP	marijuana, smoke, use, smoking, sleep, rem, smoked, marijuana use, evergreen, month, percent, days, pot, in the past, alcohol, your marijuana use, attainment, impact, we asked you, reducing, how if at, drink, class, t h c, the past, in the last, memory, drug, ability, data
Background	Background Label 1	week, days, couple, i mean, most, sorry, it’s not, a couple, drink, trying to, nights, seeing, you know, don’t think, quarter, difference, positive, kind of, compared, i mean you, month, a week, hanging, question, start, survey, the last, it just, hit, thinking about
	Background Label 10	drinking, a little bit, drink, a little, kind of, alcohol, little bit more, drinks, you’re not, buzz, a lot, need, less, stop, a lot of, you do, bit more, it’s just, sort of, starting, side, the point, of what, friends, blacking, important, relaxed, effects, question, start

T, Therapist; C, Client.

The background topics and the intervention study topics play an important role in predicting MISC codes. Specifically, these topics explain word usage that is unrelated to the behavior targeted by the MISC codes. For example, the topics associated with different types of intervention studies capture the words typical of those studies (*e.g.*, ‘marijuana’ for the iCHAMP study which focused on marijuana use, ‘birthday’ for the ESP21 study, which focused on reducing alcohol abuse during 21^st^ birthday celebrations). Similarly, the background topics capture variations in word usage that are neither explained by the type of intervention study or the MISC codes. For example, background topics 1 and 10 capture word usage related to the time and amount of drinking. Without these background and intervention study topics, high-frequency words such as ‘birthday’ or ‘marijuana’ would have to be explained by the MISC coding topics, decreasing the generalizability of those topics and the accuracy of the model in predicting MISC codes.

### Predictive performance

The labeled topic model’s predictive performance relative to human raters was evaluated via three separate comparison tasks: a comparison of a continuous prediction from the model against a rater’s code assigned at a talk turn, via receiver operating characteristic (ROC) curves; a comparison of agreement (Cohen’s Kappa) of the most likely code predicted by the model against a rater’s code for each talk turn; and a comparison of the total number of model-based codes compared to the total number of human rater codes for an entire session.

**Figure 1:**
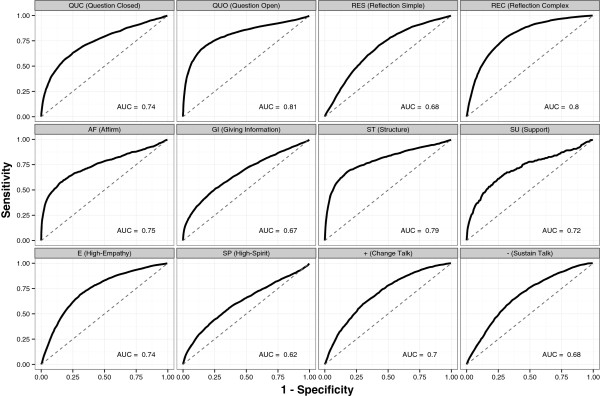
**Receiver operating characteristic curves for the set of 12 Motivational Interviewing Skills Code (MISC) codes.** AUC, Area Under Curve.

ROC curves explore the trade-off between sensitivity (true positive rate) and specificity (true negative rate; 1 – false positive rate) at various decision thresholds. Sensitivity measures the proportion of talk turns in which the model predicted a code, and the coder applied the code as well. The false alarm rate (1 – specificity) measures the proportion of talk turns in which the model predicted a code but the coder did not apply the code. The area under the ROC curve (AUC) is a useful summary statistic of discriminative performance, varying between 0.5 (chance prediction) to 1 (perfect prediction). Therefore, the AUC measures the degree to which the labeled topic model can discriminate between talk turns where the code is present or absent. Figure 1 shows ROC curves and AUC statistics for the set of 12 MISC codes. The AUC is generally above 0.5, indicating that the model reliably performs better than chance, with variation across codes. The best performance is observed for open and closed questions (QUO and QUC), complex reflections (REC), affirmations (AF), structure (ST), and empathy (E).

While the AUC statistic provides useful insights about the overall performance of the model independent of any coding bias, it does not provide a direct comparison to human reliability. For this purpose, we examined two comparisons between model-based predictions and human ratings. The first comparison focuses on talk turns and compared the average pairwise agreement (Cohen’s kappa) among human coders (rater kappa) with the average pairwise agreement of the model and each coder (model kappa). For both research and clinical purposes, a typical use of MISC codes focuses on total numbers of codes for an entire session. Thus, the second comparison between the labeled topic model and human raters examined session code totals, using the intraclass correlation coefficient (ICC) as a measure of agreement [21].

**Figure 2:**
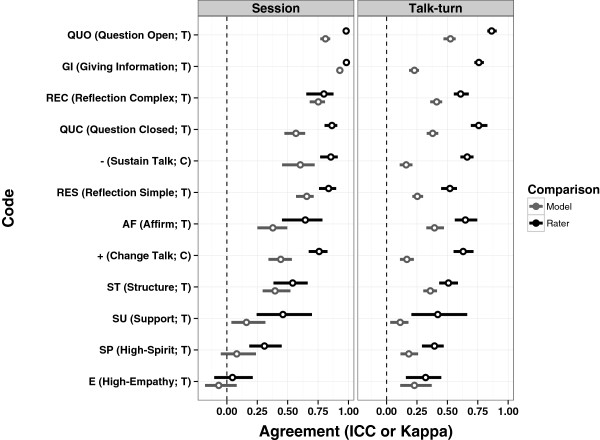
**Agreement of human raters and model raters.** Session, reliability based on sums of codes across the entire session; Talk Turn, reliability based on unique codes in each talk turn.

Figure 2 presents human rater reliability (*‘*rater*’*) and model versus human reliability (*‘*model*’*) for individual codes at the talk turn level (right-hand panel) and for session tallies of codes (left-hand panel) and is ordered from best to worst reliability overall. All comparisons were based on sessions with multiple human raters so that both human-human (*i.e.*, inter-rater) and model-human reliability could be estimated. Both human raters and model-based predictions show a wide range of reliability across codes, which is common with MISC codes [22]. Codes with more reliable semantic structure (*e.g.*, questions and reflections) generally have higher reliability than those representing more abstract interpersonal processes (*e.g.*, empathy).

Comparing the model-based predictions to human-human reliability, the labeled topic model performs significantly better than chance guessing (indicated by dotted line at zero) and is generally closer to human reliability when scores are tallied across sessions, rather than at each individual talk turn (*i.e.*, a comparison of left-hand and right-hand panels). Another important feature of the current comparisons is that the reliability of human raters represents an upper bound for the model-based reliability estimates (due to the fact that the human ratings have measurement error in them). Hence, in several cases, the topic model approach is strongly competitive with human raters (*e.g.*, complex reflections, giving information, simple reflections). Two of the 12 codes relate to patient behaviors (change talk and sustain talk). The results here suggest that the topic model has a challenging time identifying patient talk turns describing the desire to (or steps toward) change or maintain their alcohol or drug use; however, when aggregating over talk turns within a session, the model is much closer to human reliability.

A final comparison of model-based and human-based codes examined several MI proficiency indices. When used for clinical training and supervision, MISC behavioral codes are often summarized in several proficiency indices, including: ratio of reflections to questions; percent open questions out of total questions; and percent complex reflections out of total reflections. When compared to human raters, model-based estimates were relatively good for reflection to question ratio (rater ICC: 0.83; model ICC: 0.66) and percent open questions (rater ICC: 0.86; model ICC: 0.68) but notably worse for percent complex reflections (rater ICC: 0.52; model ICC: 0.10). The lower ICC for percent complex reflections relates directly to common model errors.

### Model-based errors

Table 3 contains example talk turns for instances in which the model correctly identified the human-based code, as well as instances in which the model made errors. We focus on some of the more common categories of MISC codes for therapists as well as patient change and sustain talk. Examining individual utterances highlights the general challenge of coding spoken language. For example, talk turns are not always complete sentences (*e.g.*, *‘*Okay so it sounds like drinking is kind of like a*’* and *‘*Were you doing this by yourself or were you*’*). Moreover, the labeled topic model is purely text-based and does not incorporate any acoustic information of the spoken language, which in some instances could be very telling (*e.g.*, *‘*So you are you do have housing now*’*). Human raters were listening to the session, and acoustic information could dramatically increase the accuracy in differentiating questions from reflections. At a broader level, the labeled topic model is quite good at identifying reflections or questions in general but has more difficulty in identifying the type of question or reflection. Closed versus open questions and simple versus complex reflections are commonly mistaken. These types of model errors contribute to the lower ICC for the MI proficiency rating of percent complex reflections.

**Table 3:** Examples of talk turns that were correctly or incorrectly classified by the labeled topic model

**Code**	**Model correctly classified talk turn**	**Rater codes**	**Model incorrectly classified talk turn**	**Rater codes**
QUC	Yeah does that surprise you.	QUC	How does that sound.	QUO
	Does that sound about right.	QUC	So you are you do have housing now.	REC, RES
	Yeah does that surprise you or does that seem like it’s.	QUC	Um medical risk psychological risks social risks and it’s a scale from one to ten and you were in a nine.	GI
	So well did you have any other questions for me or.	QUC	So where do you think you go from here then at this point.	QUO
	
QUO	So what do you make of that.	QUO	Were you doing this by yourself or were you.	QUC
	What do you mean by that.	QUO	Do you think you’ve hit rock bottom now.	QUC
	What do you mean by absurd.	QUO	Are you thinking of mike’s hard.	QUC
	What do you mean by that what.	QUO	In terms of your actual blood alcohol level.	QUC
	
RES	Okay and you said that you used to drink a little bit more last year it sounds like.	RES	Okay so by cutting down it sounds like you were thinking use occasionally but not use as much.	REC
	Yeah you mentioned that and you felt like that kinda.	RES	You’re talking about you and your experience.	GI, REC
	You mentioned that maybe right here with legally intoxicated I know you mentioned earlier that you always have safe transportation so that would be a more safe area for you.	GI, RES	Well it sounds like you use it in a way that’s pretty controlled.	REC
	It sounds like it it sounds like you’re doing it it sounds like you have um you say you feel better right is that physically and emotionally.	AF, QUC, REC, RES	So when you talked about going to treatment twelve times you were talking about a a or you were talking about in-patient treatment ah.	QUC
	
REC	Mm-hmm so you’re two birds of a feather it sounds like.	REC	And yet it sounds like you do have depression at times you.	RES
	Yeah it sounds like you’ve turned it around.	REC	Okay so it sounds like drinking is kind of like a.	RES
	So it really truly to me it sounds like you’ve really been thinking about this pretty hard.	REC	And it seems like you’re not having any withdrawals at this point but you’ve physically or psychologically dependent upon alcohol.	GI, QUC, RES
	Well it sounds like that’s a big deterrent for you.	REC	That’s hard it sounds like but you know what but.	GI, RES, SU
	
+	I put alcohol by myself I can do anything.	+	You know because I know I’m a good person.	FN
	Doing anything with that coke that scares me that straight up scares me.	+	But I really don’t know that I had a choice.	-
	Because it’s not good for you and it’s bad and I don’t like it it makes me feel guilty and.	+	Yeah but I I just can’t leave the cocaine alone I can’t stay away from it.	-
	Well I shouldn’t be doing it and it’s illegal it’s it’s bad for you I know it's bad and I do it because when I drink too much sometimes I do that because it’s so much it’s all around me not all around me but it’s easy accessible to get and when I’m not drinking I would never touch it.	+, -	Just like I said my own excitement.	-
	
-	Yeah I don’t feel like I drink anything crazy or anything besides strictly beer I’m pretty cheap too so.	+, -	I usually do mix when I drink actually.	FN
	Yes I do smoke weed and I would smoke weed every day if I had it I mean I used to when I was younger I used to but now I only smoke it when I’m around certain people and I only see them maybe once a week so to me that’s not severe or heavy.	-	Feel stressed I take a lot of naps when I get stressed out and watch movies stuff like that back home I take baths but I can’t really do that here so.	FN
	Yes if I have to only if I have to I change but if I don’t have to I can survive working there working here I’m not gonna quit man.	+, -	It kind of explains a lot because I take naps just about every day and like I don’t usually dream at night but when I take naps I have some pretty intense dreams.	+
	Like I said I mean it’s been a while since I really had a problem.	-	Right absolutely it means not it’s not a it’s I’m probably underestimating what I feel I’m probably lowballing how horrible I feel honestly.	FN

QUC, Therapist closed question; QUO, Therapist open question; REC, Therapist complex reflection; RES, Therapist simple reflection; AF, Therapist affirmation; GI, Therapist giving information; SU, Therapist support; FN, Client follow/neutral; + , Client change talk; -, Client sustain talk.

## Discussion

The technology for evaluating psychotherapy has remained largely unchanged since Carl Rogers first published verbatim transcripts in the 1940s: sessions are recorded and then evaluated by human raters [23,24]. Given the sheer volume of behavioral interventions in the healthcare delivery system, human evaluation will never be a feasible method for evaluating provider fidelity on a large scale. As a direct extension of this, feedback is rarely available to substance abuse providers in the community, and thus, therapists typically practice in a vacuum with little or no guidance on the quality of their therapy [25]. Similarly, clinic administrators have no information about the quality of their psychotherapy services.

The present research provides initial support for the utility of statistical text classification methods in the evaluation of psychotherapy. Using only text input, the labeled topic model showed a strong degree of accuracy for particular codes when tallied over sessions (*e.g.*, open questions, giving information, and complex reflections) and was similar to human rater reliability in several other instances (*e.g.*, simple reflections, structure). Moreover, summary agreement statistics did not always reveal how near or far the model was from human accuracy. For example, model performance for closed questions was notably below human raters; however, the most common code that the model confused with closed questions was open questions. On the one hand, further work should be done to improve the model’s ability to discriminate closed and open questions, yet the model is clearly identifying some of the critical lexical information in questions generally. The model was far less accurate compared to human ratings at the talk turn level, perhaps suggesting that the limited information in talk turns needs to be augmented with additional local context to improve accuracy. Another possibility is that the measurement error in individual, human codes leads to poorer model performance for talk turns, but when evaluated as tallies for a whole session, the measurement error is averaged out and comparisons are more reliable.

The predictive performance of the model would appear to directly relate to the linguistic (and specifically, lexical) basis of the codes. Some of the current behavioral codes are strongly linguistic in nature, such as questions and reflections. There are characteristic words and short phrases that are emblematic of such speech. In these areas, the labeled topic model was strongly competitive with human raters, whereas for codes that are more abstract, such as empathy and *‘*MI spirit*,’* both humans and the labeled topic model had challenges. With an eye toward applying the current methodology to other coding systems, behavioral codes that have a strong lexical element should be good candidates for the current methods (*e.g.*, reviewing homework in cognitive behavioral therapy, identifying specific types of cognitive distortions).

This study represents a preliminary step toward developing an automated coding system. Typical behavioral coding is onerously time-consuming and error prone, presenting a barrier to the evaluation of disseminated treatments and research on the specific psychological mechanisms responsible for patient response. For example, following approximately three months of training, the MISC coding of the current data took approximately nine months to generate with three part-time coders. The long-term goal of the current research is to develop a system that would take audio input and yield codes, and preliminary work on the speech signal processing aspects of such a system is already underway [26,27]. With a computational system, reliability between raters would be removed outside of periodic calibration checks of model to humans. Concerns about coder drift and training new coders would also be reduced.

One clear application for the current methodology is in the large-scale evaluation of disseminated interventions in naturalistic settings, which is primarily a clinical focus. However, there is also a clear research application as well. There are promising findings from observational coding studies that suggest MI-consistent provider behaviors result in positive treatment outcomes [19,28,29], though this remains a relatively small literature. Outside of MI, the literature examining the relationship of therapist adherence ratings with patient outcomes is limited. A recent meta-analysis included 36 studies with an average sample size of 40 patients per study [8]. The average correlation of therapist quality and patient outcomes across studies was close to zero, but effects were highly variable, ranging from –0.40 to 0.73) likely attributable to the small, per-study sample sizes. Computational models would greatly increase the size of these studies and may result in dramatically more powerful studies of treatment mechanisms in psychotherapy.

There are also several clear extensions that could be explored for enhancing the model and its predictive ability with behavioral codes. First, the labeled topic model tested in the current research does not make use of the ordering of talk turns, whereas other research has shown that the local context of an utterance (*i.e.*, what comes immediately before and after) can be important for accurate code prediction [30]. This may be particularly important for differentiating simple from complex reflections. The former often show strong similarity with the preceding, patient talk turn, whereas the latter often incorporate content from a broader portion of the session. Second, human raters typically generate their ratings based on audio (and sometimes video) recordings, and codes often have distinctive tonal features (*e.g.*, increasing intonation in questions, or decreasing in confrontations). Research has shown that acoustic features alone can be indicative of some behavioral codes [31]. Third, the current methods require transcripts, which is a potential limitation for their proposal to scale up coding of behavioral interventions. The technology of speech recognition continues to improve and some research has shown that lexical models can be successfully based off automated speech recognition inputs [27]. Each of the above represents future directions that can build off the current work, though all would focus on replicating a human-derived coding system (the MISC system in the present study). A final, more speculative, future direction would be pursuing research that might extend beyond simply replicating human-based provider fidelity coding systems. That is, could topic models and other machine learning approaches discover semantic and acoustic features within therapy sessions that are not currently coded but are related to improved patient outcomes? While a clear strength of human-derived coding systems is the distillation of treatment developer and clinician expertise, it is also possible that by focusing on specific provider behaviors and interactional patterns other important behaviors and patterns are missed.

## Conclusions

The current research demonstrated preliminary support for using statistical text classification to automatically code behavioral intervention sessions. The current method of evaluation using human raters will never scale up to meaningful clinical use and a technological solution is needed. Technology has strongly influenced many of our basic tasks of daily living (*e.g.*, cell phones, internet search), including automated text analysis (*e.g.*, spam email classification, automated news summarization, sentiment classification in product reviews). A similar, technological transformation is needed in psychotherapy, an intervention that is essentially a conversation, but difficult to measure. The current research and findings represent an initial step in that direction.

## Competing interests

All authors declare that they have no competing interest.

## Authors’ contributions

All authors had full access to all the data in the present study and take responsibility for the integrity of the data and accuracy of results. DCA contributed to general conception of study, design, and interpretation of findings. MS helped with design and conducted all statistical analyses. ZI contributed to original conception and interpretation of findings. PS assisted with analyses and interpretation. All authors contributed to writing and editing the manuscript.

## Supplementary Material

Additional file 1 Supplemental Appendix. Click here for file

## References

[B1] Substance Abuse and Mental Health Services Administration, Center for Behavioral Health Statistics and Quality Treatment Episode Data Set (TEDS): 2000-2010. State Admissions to Substance Abuse Treatment Services DASIS Series: S-63 2012 SMA-12-4729 Rockville, MD: Substance Abuse and Mental Health Services Administration http://www.samhsa.gov/data/2k13/TEDS2010/TEDS2010StTOC.htm

[B2] Sox-Harris A Gifford E Hagedom H Ekstrom J Substance Use Disorder QUERI: Strategic Plan 2011 Palo Alto, CA: VA Palo Alto Health Care System http://www.queri.research.va.gov/about/strategic_plans/sud.pdf

[B3] Miller WR Wilbourne PL Mesa Grande: a methodological analysis of clinical trials of treatments for alcohol use disorders Addiction 2002 97 265 277 10.1046/j.1360-0443.2002.00019.x 11964100

[B4] Miller WR Rose GS Toward a theory of motivational interviewing Am Psychol 2009 64 527 537 19739882 10.1037/a0016830 PMC2759607

[B5] Department of Veteran Affairs Practice Recommendations for Treatment of Veterans with Comorbid Substance Use Disorder and Posttraumatic Stress Disorder 2009 http://www.mentalhealth.va.gov/providers/sud/docs/SUD_PTSD_Practice_Recommendations.pdf

[B6] Proctor E Silmere H Raghavan R Hovman P Aarons G Bunger A Griffey R Hensley M Outcomes for implementation research: conceptual distinctions, measurement challenges, and research agenda Adm Policy Ment Health 2011 38 65 76 10.1007/s10488-010-0319-7 20957426 PMC3068522

[B7] Glynn LH Hallgren KA Houck JM Moyers TB CACTI: free, open-source software for the sequential coding of behavioral interactions PLoS One 2012 7 7 e39740 doi: 10.1371/journal.pone.0039740 10.1371/journal.pone.0039740 22815713 PMC3397966

[B8] Webb CA DeRubeis RJ Barber JP Therapist adherence/competence and treatment outcome: A meta-analytic review J Consult Clin Psychol 2010 78 200 211 20350031 10.1037/a0018912 PMC4246504

[B9] Blei D Probabilistic topic models Comm ACM 2012 55 4 77 84 10.1145/2133806.2133826

[B10] Blei D Ng AY Jordan MI Latent Dirichlet allocation J Mach Learn Res 2003 3 993 1022

[B11] Griffiths TL Steyvers M Finding scientific topics Proceedings of the National Academy of Sciences of the United States of America 2004 **101**:5228-5235 10.1073/pnas.0307752101 PMC387300 14872004

[B12] Steyvers M Griffiths T Landauer T, McNamara D, Dennis S, Kintsch W Probabilistic topic models Latent Semantic Analysis: A Road to Meaning 2006 Mahwah, NJ: Erlbaum 427 448

[B13] Atkins DC Rubin TN Steyvers M Doeden M Baucom B Christensen A Topic models: a novel method for modeling couple and family text data J Fam Psychol 2012 26 816 827 22888778 10.1037/a0029607 PMC3468715

[B14] Rubin T Chambers A Smyth P Steyvers M Statistical topic models for multi-label document classification J Mach Learn 2012 88 1 157 208

[B15] Krupski A Joesch JM Dunn C Donovan D Bumgardner K Lord SP Ries R Roy-Byrne P Testing the effects of brief intervention in primary care for problem drug use in a randomized controlled trial: rationale, design, and methods Addict Sci Clin Pract 2012 7 1 27 10.1186/1940-0640-7-27 23237456 PMC3598998

[B16] Lee CM Kilmer JR Neighbors C Atkins DC Zheng C Walker DD Larimer ME Indicated prevention for college student marijuana use: A randomized controlled trial J Consult Clin Psychol 2013 81 4 702 709 23750464 10.1037/a0033285 PMC3924720

[B17] Lee CM Neighbors C Lewis MA Kaysen D Mittman A Geisner IM Atkins DC Zheng C Garberson LA Kilmer JR Larimer ME Randomized controlled trial of a spring break intervention to reduce high-risk drinking J Consult Clin Psychol 2014 82 2 189 201 24491072 10.1037/a0035743 PMC4390296

[B18] Neighbors C Lee CM Atkins DC Lewis MA Kaysen D Mittmann A Fossos N Geisner IM Zheng C Larimer ME A randomized controlled trial of event-specific prevention strategies for reducing problematic drinking associated with 21st birthday celebrations J Consult Clin Psychol 2012 80 5 850 862 22823855 10.1037/a0029480 PMC3458124

[B19] Tollison SJ Lee CM Neighbors C Neil TA Olson ND Larimer ME Questions and reflections: The use of motivational interviewing microskills in a peer-led brief alcohol intervention for college students Behav Ther 2008 39 2 183 194 10.1016/j.beth.2007.07.001 18502251 PMC5361059

[B20] Miller WR Moyers TB Ernst DB Amrhein PC Manual for the Motivational Interviewing Skill Code (MISC), Version 2.1 2008 New Mexico: Center on Alcoholism, Substance Abuse, and Addictions, The University of New Mexico

[B21] Shrout PE Fleiss JL Intraclass correlations: Uses in assessing rater reliability Psychol Bull 1979 86 420 428 18839484 10.1037//0033-2909.86.2.420

[B22] Forsberg L Kallmen H Hermansson U Berman AH Helgason AR Coding counselor behavior in motivational sessions: Inter-rater reliability for the Swedish Motivational Interviewing Treatment Integrity Code (MITI) Cognit Behav Ther 2007 36 3 162 169 10.1080/16506070701339887 17852172

[B23] Kirschenbaum H Carl Rogers’s life and work: an assessment on the 100th anniversary of his birth J Counsel Dev 2004 82 1 116 124 10.1002/j.1556-6678.2004.tb00293.x

[B24] Rogers CR Counseling and Psychotherapy: Newer Concepts in Practice 1942 Boston: Houghton Mifflin

[B25] Miller W Sorensen J Selzer J Disseminating evidence-based practices in substance abuse treatment: a review with suggestions J Subst Abuse Treat 2006 31 25 39 10.1016/j.jsat.2006.03.005 16814008

[B26] Can D Gibson J Vaz C Georgiou PG Narayanan SS Barista: A Framework for Concurrent Speech Processing from USC-SAIL Proceedings of IEEE International Conference on Audio, Speech, and Signal Processing (ICASSP): 4-9 May 2014 Florence, Italy: Institute of Electrical and Electronics Engineers http://ieeexplore.ieee.org/xpl/conhome.jsp?punumber=1000002 10.1109/ICASSP.2014.6854212 PMC5012110 27610047

[B27] Georgiou PG Black M Lambert A Baucom B Narayanan SS That’s Aggravating, Very Aggravating: Is it Possible to Classify Behaviors in Couple Interactions Using Automatically Derived Lexical Features? Proceedings of Affective Computing and Intelligent Interaction: 9-12 October 2011; Memphis, TN 2011 Berlin, Heidelberg: Springer-Verlag 87 96

[B28] Apodaca TR Longabaugh R Mechanisms of change in MI: a review and preliminary evaluation of the evidence Addiction 2009 104 705 715 10.1111/j.1360-0443.2009.02527.x 19413785 PMC2756738

[B29] Moyers TB Martin T Christopher PJ Houck JM Tonigan JS Amrhein PC Client language as a mediator of motivational interviewing efficacy: Where is the evidence? Alcohol Clin Exp Res 2007 31 Suppl 3 40S 47S 17880345 10.1111/j.1530-0277.2007.00492.x

[B30] Can D Georgiou PG Atkins DC Narayanan SS A Case Study: Detecting Counselor Reflections in Psychotherapy for Addictions Using Linguistic Features 2012 Paper presented at the Annual Conference of the International Speech Communication Association: 9-13 September 2012; Portland, OR

[B31] Black MP Katsamanis A Baucom BR Lee CC Lammert AC Christensen A Georgiou PG Narayanan SS Toward automating a human behavioral coding system for married couples’ interactions using speech acoustic features Speech Comm 2013 55 1 21 10.1016/j.specom.2011.12.003

