# Supplementary material for: Scaling up the evaluation of psychotherapy: evaluating motivational interviewing fidelity via statistical text classification
Source: Implement Sci. 2014 Apr 24;9:49. doi: 10.1186/1748-5908-9-49 (PMC4026152; doi:10.1186/1748-5908-9-49)
Supplement: Additional file 1 — Supplemental Appendix. [file 1748-5908-9-49-S1.docx]

# Supplemental Appendix (to accompany: Scaling up the evaluation of psychotherapy)

In this Appendix, we provide some additional methodological detail on the preprocessing of the therapy transcripts, labeled topic model, and calculation of reliability scores. Throughout the appendix we highlight additional resources for those readers wanting greater depth on particular topics.

## Preprocessing of Therapy Transcripts

The linguistic representation in our analysis focuses on the set of terms called n-grams in each talk turn, where a term consists of individual words (unigrams) as well as combinations of words involving two or three words (bigrams and trigrams). The present analysis ignores the order of the terms in a talk turn -- only the frequency of terms in a talk turn is considered. This information is captured in a matrix, where each cell represents the total number of times a term appears in a particular talk turn. The talk turn level was considered an appropriate level of granularity to aggregate the word counts, though word counts at a smaller level of granularity such as utterances (complete thoughts) could be considered.

In constructing the term counts, we removed terms corresponding to very high frequency words (e.g., “the”, “to”, “and”) that did not provide any useful semantic information; see Atkins et al.^1^ for details. Furthermore, we used a part-of-speech tagger^2^ to filter out specific combinations of n-grams that do not correspond to natural linguistic units (e.g. n-grams ending in conjunctions or determiners such as *Spring Break and*). In addition, we excluded low frequency terms that occurred fewer than 5 times in total. After preprocessing, the vocabulary consisted of approximately 3,000 unigrams, 15,000 bigrams, and 15,000 trigrams.

## Coding of Talk Turns

A modified version of the MISC 2.1^3^ was used to code each transcript^4^. In the original coding of the data, each coder segmented talk turns into utterances (i.e., complete thoughts) and assigned one code per utterance for all utterances in a session. For each talk turn, we created a binary coding scheme in the following way. For the sessions coded by a single coder (79%; *n*= 117), the codes at a particular talk turn were defined as the unique codes across utterances within that talk turn (ignoring the repeat codes). For the sessions coded by three coders (21%; *n*=31), the codes at the talk turn were defined as the unique codes across the utterances within a talk turn across all three coders (ignoring repeat codes across coders and utterances). Therefore, in this binary coding scheme, a code is present if any of the coders tagged the code to any of the utterances in the talk turn.

In order to analyze codes for which reliable information was available, we excluded six codes that were present in fewer than 2% or more of talk-turns leaving a subset of 12 codes. Table A1 shows the 12 codes with their relative frequency.

**Table A1. Relative Frequency of Codes across Talk Turns**

| **Code^a^** | **% Talk Turns** |
| --- | --- |
| QUC (Question Closed; T) | 25 |
| QUO (Question Open; T) | 30 |
| RES (Reflection Simple; T) | 32 |
| REC (Reflection Complex; T) | 30 |
| AF (Affirm; T) | 11 |
| GI (Giving Information; T) | 48 |
| ST (Structure; T) | 10 |
| SU (Support; T) | 2 |
| E (High-Empathy; T) | 30 |
| SP (High-Spirit; T) | 31 |
| + (Change Talk; C) | 23 |
| - (Sustain Talk; C) | 21 |
| ^a^ Descriptions of codes are given between parentheses; T=Therapist Code; C=Client Code | |

## Labelled Topic Model

The topic model in our analysis is closely related to the labeled LDA model^5^, the flat-LDA model in Rubin et al.^6^, and the concept-topic model^7,8,9^. In our approach, we applied the labeled topic model to the session transcripts and treated the talk turn as the unit of analysis (i.e., corresponding to a “document” in the language of topic models; see Atkins et al.^1^, for general introduction to topic models for a psychology audience). The learning algorithm iteratively adjusts the parameters of the topic-word and document-topic distributions in a Bayesian framework to optimize the fit of the topic model to the observed words and codes – see Griffiths and Steyvers^10^ for details.

## Model Evaluation Procedures

As noted in the text, the model was trained and tested using a 10-fold cross-validation procedure at the level of sessions. The 148 sessions were divided into 10 (roughly equal size) subsets of sessions, and then the model is iteratively fit to 90% of the sessions and tested on the 10% of sessions that were withheld. This is repeated 10 times and final results are averaged over the 10 runs.

The training data for the model consisted of the term counts for each talk turn in the training sessions. In addition, the model was provided with the topics associated with each talk turn. A subset of topics corresponded directly to the 12 behavioral codes. An additional set of topics was included to encode information about the type of intervention study – we allowed five topics for this purpose, one each for the five intervention studies in our corpus. In addition, a subset of background topics were included that correspond to latent semantic clusters, which are very similar to the typical usage of topic models with text that does not include codes or other types of meta-data. The idea is that these additional topics allow the model to capture some of the linguistic variability in the data that is not directly related to the behavioral codes. For example, the topics that encode the type of study can explain the word usage that might be pervasive in particular intervention studies. The additional latent topics can model word usage that is neither explained by the type of study (or any other feature used from the meta-data) nor the behavioral codes. Without these additional topics many words would have to be explained by the topics associated with the behavioral codes, decreasing the generalizability of those topics.

For the sessions in the test set (i.e., 10% of sessions that were withheld), the model was provided with the linguistic information in each talk turn (term counts). The model was also provided information about the topics associated with the type of intervention study. While the model was not provided with the actual codes for the talk turns in the test sessions, it was given information about the logical restrictions placed on the codes based on the speaker information. For example, if a talk turn was associated with the client, patient codes were not allowed to be expressed by the model.

For each (allowable) code in a talk turn in a test session, the model computed a continuous score that captures how relevant the code is given the linguistic information contained in the talk turn. In order to compare the model predictions to the human codes, and calculate Kappa and ICC reliability indices, the continuous model scores were binarized by comparing the scores to a threshold. The thresholds were determined separately for each code and each coder. Specifically, to calculate a reliability score between the model and a particular human coder (say, coder A), the threshold for a particular code was chosen such that the model produced the same number of codes as one of the *other* human coders (either coder B or C). Therefore, the thresholds were optimized such that the model matched the base rate of a human coder, but not for the particular human coder it was compared to. In total, we calculated six model reliability values (the model predictions were compared to each of the three raters with thresholds optimized to each of the two other raters). The Figures in the main document show the average reliability scores across these six values.

## Confidence Intervals

A bootstrap procedure was used to calculate the 95% confidence intervals in Figure 2. For the model-based Kappa and ICC reliability indices, the model’s binary predictions calibrated to particular human coder were compared to another human coder separately for each test session. For each specific pairwise comparison, we drew 1,000 bootstrap samples of the Kappa or ICC values across sessions, reflecting the variability in the mean of Kappa or ICC across sessions. The 95% confidence intervals were then computed on the bootstrap samples concatenated across the six pairwise comparisons of model to human coder. For human-coder based Kappa and ICC reliability indices, a similar procedure was used but based on the three pairwise comparisons between the three human coders.

References

1. Atkins DC, Rubin TN, Steyvers M, Doeden M, Baucom B, Christensen A. Topic models: A novel method for modeling couple and family text data. *Journal of Family Psychology.* 2012;26:816-827.
2. Toutanova T, Klein D, Manning C, Singer, Y. Feature-rich part-of-speech tagging with a cyclic dependency network. In *Proceedings of HLT-NAACL.* 2003:252-259.
3. Miller WR, Moyers TB., Ernst DB, Amrhein PC. *Manual for the Motivational Interviewing Skill Code (MISC), Version 2.1*. New Mexico: Center on Alcoholism, Substance Abuse, and Addictions, The University of New Mexico. 2008.
4. Lord SP, Can D, Yi MSK, Marín RA, Dunn CW, Georgiou PG, Narayanan SS, Steyvers M, Atkins DC. *Utterances vs. sessions: Novel methods of collection and reliability for the Motivational Interviewing Skills Code.* Manuscript under review. 2013.
5. Ramage D, Hall D, Nallapati R, Manning CD. Labeled LDA: A supervised topic model for credit attribution in multi-labeled corpora. In *Proceedings of the 2009 conference on empirical methods in natural language processing*, Singapore, August 2009 (pp. 248–256). Association for Computational Linguistics.
6. Rubin T, Chambers A., Smyth P, Steyvers M. Statistical Topic Models for Multi-Label Document Classification. *Journal of Machine Learning. 88*(1);2012:157-208.
7. Chemudugunta C, Smyth P, Steyvers M. Combining concept hierarchies and statistical topic models. *Conference on Information and Knowledge Management (CIKM)*. Napa Valley, CA October 2008
8. Chemudugunta C, Smyth P, Steyvers, M. Text modeling using unsupervised topic models and concept hierarchies. *ArXiv Technical Report*. 2008:1-22. url: http://arxiv.org/abs/0808.0973
9. Steyvers M, Smyth P, Chemudugunta C. Combining background knowledge and learned topics. *Topics in Cognitive Science*. 2011;3(1):18-47.
10. Griffiths T, Steyvers M. Finding scientific topics. Proceedings of the National Academy of Sciences. 2004;101(suppl. 1):5228-5235.
